# *Aspergillus fumigatus* native valve infective endocarditis in an otherwise healthy adult

**DOI:** 10.1099/jmmcr.0.005018

**Published:** 2016-02-18

**Authors:** Uchenna Ikediobi, Richard E. Sutton

**Affiliations:** Section of Infectious Diseases, Department of Internal Medicine, Yale School of Medicine, New Haven, CT 6520, USA

**Keywords:** *Aspergillus fumigatus*, fungal endocarditis, native valve endocarditis

## Abstract

**Introduction:**

Fungal endocarditis is a rare cause of infective endocarditis, and *Aspergillus* spp. account for up to 30 % of all cases. Risk factors include intravenous drug use, immunosuppression, malignancy and the presence of prosthetic valves.

**Case presentation:**

We present a case of *A. fumigatus* endocarditis in a patient without any known or described risk factors.

**Conclusion:**

Diagnosis of *Aspergillus* endocarditis requires a high clinical index of suspicion, given the initial non-specific presentation, and treatment may require both medical and surgical therapies to ensure improved outcomes, but mortality rates still approach 80 %. Voriconazole remains the antifungal agent of choice.

## Introduction

Fungal endocarditis accounts for less than 10 % of all infective endocarditis cases with *Aspergillus* spp. accounting for up to 30 % of all cases (Kalokhe *et al.*, 2010). The risk factors associated with fungal endocarditis include intravenous drug use, immunosuppression, malignancy and the presence of prosthetic valves (Kalokhe *et al.*, 2010; Ellis *et al.*, 2001, Pierrotti & Baddour, 2002).

Here, we present a case of *Aspergillus fumigatus* endocarditis in a patient without any known or described risk factors.

## Case report

A 65-year-old healthy man presented to a local hospital with a 3-month history of chest pain, dyspnoea and fatigue. He was a retired school teacher with no appreciable history of intravenous drug use, recent steroid or other immunosuppressive medication exposures, malignancy, or any known congenital or acquired cardiac disease. He had a history of an ill-defined heart murmur since childhood but no prior history of invasive fungal or bacterial infections. He denied any prior surgeries, including any cardiovascular procedures or implantation of cardiac devices. Initial evaluation revealed an electrocardiogram showing Mobitz type II AV heart block and a new left bundle branch block. Transthoracic and transoesophageal echocardiograms demonstrated a 1.6 × 1.2 cm vegetation on the anterior cusp of a trileaflet aortic valve, mild to moderate aortic regurgitation and a medium-sized sessile vegetation (dimensions not reported) on the mitral valve, with mild mitral regurgitation. There was no other valvular pathology. Two sets of blood cultures were negative. He was started on intravenous ampicillin-sulbactam and gentamicin for suspected culture-negative endocarditis and transferred to Yale-New Haven Hospital.

On arrival at the hospital, his vital signs were: temperature of 100.4 °F, heart rate of 84 beats min^− 1^, respiratory rate of 20 min^− 1^, blood pressure of 130/70 mmHg and oxygen saturation of 98 % (room air). Physical examination revealed a non-toxic appearing man with a grade 3/6 systolic murmur heard best at the right upper sternal border, with no appreciable radiation or signs of heart failure or stigmata of infective endocarditis.

The laboratory data were remarkable for a white blood cell count of 12.7 × 10^9^ l^− 1^ (normal range 4.5 − 11.0 × 10^9^ l^− 1^), an erythrocyte sedimentation rate of 114 mm h^− 1^ (normal range 0–22 mm h^− 1^), seronegativity for human immunodeficiency virus type 1 and 2 antibodies, and levels of IgG of 1050 mg dl^− 1^, IgA of 254 mg dl^− 1^ and of IgM 59 mg dl^− 1^ (all within normal limits). Extensive review of the laboratory and radiological data, including a haemogram, metabolic profile and computed tomography of the chest, did not suggest the presence of underlying immunosuppression or other co-morbidities. Noted on the computed tomography, however, were small mediastinal lymph nodes. A urine drug screen was not performed. The electrocardiogram showed a first-degree AV block and a left bundle branch block. Two sets of blood cultures were negative.

**Fig. 1:**
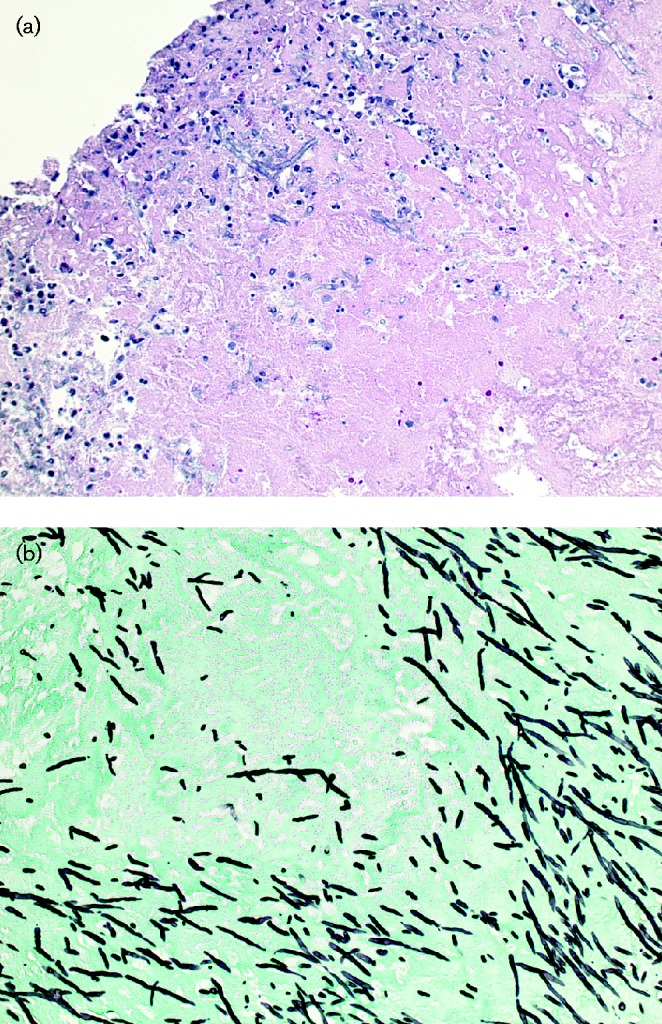
Histopathology of valvular tissue. (a) Haematoxylin and eosin stain of aortic valve tissue showing acute and chronic inflammation and myxoid degeneration, and what appeared to be hyphal elements. (b) Gomori methenamine silver stain of fixed and sectioned aortic valve tissue showing acute branching of septate hyphae consistent with *Aspergillus* spp.

He underwent radical reconstruction of the aortic root, mitral valve replacement with a 25 mm Magna ThermaFix mitral valve and aortic valve replacement with a 19 mm Magna ThermaFix aortic valve. Mediastinal lymph nodes were sent for histology and were reactive and negative for malignancy. Extracted aortic valve and mitral valve tissues were sent for culture and grew *A. fumigatus.* Valvular specimens stained with haematoxylin and eosin and Gomori methenamine silver stains showed tissue-invasive organisms morphologically consistent with *Aspergillus* spp. (Fig. 1). The patient was started on intravenous voriconazole. Three days later, a transoesophageal echocardiogram revealed mild unseating of the mitral valve prosthesis and an aortic valve with severe perivalvular regurgitation and stenosis. He underwent radical reconstruction of the left ventricular outflow tract and reimplantation of the 19 mm Magna ThermaFix aortic valve. He completed 4 weeks of dual antifungal therapy with intravenous voriconazole and amphotericin B and was discharged home on life-long oral voriconazole.

He presented 2 months later with dyspnoea on exertion and was found to have bilateral pleural effusions. A repeat transoesophageal echocardiogram showed new dehiscence of the aortic valve from the previously placed patch, new significant perivalvular aortic regurgitation and an increase in the aortic valve pressure gradient. He underwent bilateral thoracentesis, which revealed sterile transudative effusions but died 3 days later from cardiogenic shock.

## Discussion

Fungal endocarditis is a rare clinical entity with *Aspergillus* spp. infection accounting for 30 % of all such cases (Kalokhe *et al.*, 2010). Susceptible hosts typically exhibit predisposing conditions such as intravenous drug use, neutropenia, haematological malignancy, immunosuppression, prior cardiac surgery or the presence of a prosthetic valve (Kalokhe *et al.*, 2010; Ellis *et al.*, 2001; Pierrotti & Baddour, 2002). These risk factors mirror those associated with invasive fungal disease, thus reflecting the host reduction in immune defences. It is unusual to develop *Aspergillus* endocarditis in the complete absence of one or more of these risk factors. Therefore, to the best of our knowledge, our report reflects the first occurrence of its kind.

Diagnosis requires a high clinical index of suspicion, given the initial non-specific presentation. Blood cultures are rarely positive, and the utility of the galactomannan assay has not been studied in infective endocarditis (Kalokhe *et al.*, 2010). Histological and tissue culture confirmation remain the gold standard for diagnosis, as was the case here (Kalokhe *et al.*, 2010).

Treatment often requires both medical and surgical therapies to ensure improved outcomes, but mortality rates still approach 80 %, despite optimal therapy (Kalokhe *et al.*, 2010). Voriconazole remains the antifungal agent of choice for *Aspergillus* endocarditis (Kalokhe *et al.*, 2010; Ellis *et al.*, 2001; Pierrotti & Baddour, 2002).
